# Reconstruction and Analysis of Gene Networks of Human Neurotransmitter Systems Reveal Genes with Contentious Manifestation for Anxiety, Depression, and Intellectual Disabilities

**DOI:** 10.3390/genes10090699

**Published:** 2019-09-11

**Authors:** Roman Ivanov, Vladimir Zamyatin, Alexandra Klimenko, Yury Matushkin, Alexander Savostyanov, Sergey Lashin

**Affiliations:** 1 Institute of Cytology and Genetics SB RAS, 630090 Novosibirsk, Russia; zamyatin@bionet.nsc.ru (V.Z.); klimenko@bionet.nsc.ru (A.K.); mat@bionet.nsc.ru (Y.M.); Alexander.Savostyanov@gmail.com (A.S.); lashin@bionet.nsc.ru (S.L.); 2 Novosibirsk State University, 630090 Novosibirsk, Russia; 3 Institute of Physiology and Basic Medicine SB RAMS, 630117 Novosibirsk, Russia

**Keywords:** intellectual disabilities, depression, neurotransmitter, gene network, SNP

## Abstract

Background: The study of the biological basis of anxiety, depression, and intellectual disabilities in humans is one of the most actual problems of modern neurophysiology. Of particular interest is the study of complex interactions between molecular genetic factors, electrophysiological properties of the nervous system, and the behavioral characteristics of people. The neurobiological understanding of neuropsychiatric disorders requires not only the identification of genes that play a role in the molecular mechanisms of the occurrence and course of diseases, but also the understanding of complex interactions that occur between these genes. A systematic study of such interactions obviously contributes to the development of new methods of diagnosis, prevention, and treatment of disorders, as the orientation to allele variants of individual loci is not reliable enough, because the literature describes a number of genes, the same alleles of which can be associated with different, sometimes extremely different variants of phenotypic traits, depending on the genetic background, of their carriers, habitat, and other factors. Results: In our study, we have reconstructed a series of gene networks (in the form of protein–protein interactions networks, as well as networks of transcription regulation) to build a model of the influence of complex interactions of environmental factors and genetic risk factors for intellectual disability, depression, and other disorders in human behavior. Conclusion: A list of candidate genes whose expression is presumably associated with environmental factors and has potentially contentious manifestation for behavioral and neurological traits is identified for further experimental verification.

## 1. Introduction

Anxiety and major depressive disorders are the most common stress-related neuropsychiatric disorders affecting approximately 10–15% of the human population [1,2]. Depressive disorders can be long-lasting, and are often accompanied by low self-esteem, loss of interest in normally enjoyable activities, low energy, pain without a clear cause, and increased risk of suicide [3]. Different forms of intellectual disability have a pronounced relationship with the risk of depression development and anxiety disorder. On the one hand, children and adults who have intellectual disability and autism often demonstrate comorbid anxiety disorder and/or depression [4,5]. On the other hand, depression and anxiety disorder are accompanied by impaired cognitive abilities in patients [6]. Many social and physical factors are associated simultaneously with depression, anxiety disorder, and intellectual disability [7]. In addition, there are data on the connection of these disorders through generations of patients. For example, according to the study of Scherer et al. [8], the parents of children with intellectual and developmental disabilities indicated elevated levels of depressive symptoms. 

Epidemiological studies show that 40–50% of risk factors for both depression and intellectual disability can be linked to individual genetics, indicating that these disorders are highly hereditary diseases. Despite the hereditary association, specific genetic risk factors, mechanisms of development, and emergence of treatment-resistant neuropsychiatric disorders are still not fully defined. There are many papers devoted to the study of the relationships between allelic polymorphisms of different genes with the peculiarities of human behavior in health and disease [4,5,6,7]. Although it is undoubtedly established that such a relationship exists and can be studied, the results are still not reliable enough to predict the characteristics of human behavior [8,9]. The search for genetic markers of neuropsychiatric disorders has revealed that there is likely to be no unambiguous relationship between genotype and risk of developing such diseases [9]. Genetic features indicate the likelihood of pathology, but this probability is modulated by many other factors, both increasing and reducing the risk of disease [10]. The search for the relationship between genetic and personal psychological characteristics in healthy people shows that such relationships are reliably detected in groups of people living in approximately the same socio-climatic conditions [11,12]. Associations between genetic and psychological characteristics identified in one of these groups may not be observed, or may even vary to the extent of inverse relationship, in other groups of people [11,13,14,15]. A similar situation is observed for the study of the relationship between neurophysiological (brain electrophysiological or hemodynamic activity), behavioral, and psychological characteristics of healthy people and clinical patients.

This is due to the fact that finding the genetic background of psychiatric diseases is accompanied by complexities, including the fact that most of them are complex diseases with many interrelated genes affecting their development. In addition, all neuropsychiatric disorders are only partially dependent on genes. Non-genetic factors, such as environmental factors and socio-economic factors, are also expected to affect people with such disorders.

Due to the lack of understanding of molecular genetic mechanisms of intellectual disability, anxiety, and depressive disorders, medicine and neurophysiology face an obstacle to the development of new approaches to the treatment and prevention of these diseases. In this study, we use the analysis of several databases of human genetic markers to identify genes associated with human behavior and/or neuropsychiatric disorders, which reveal an ambiguous and contentious relationship between the genotype and phenotype. On the basis of this sample we reconstruct networks of protein–protein interactions and networks of transcriptional regulation. Genes added to the networks can be used as an extended test panel to identify the relationship between allele polymorphisms, indicators of psychological tests, and other behavioral traits, as well as the traits of neuropsychiatric disorders. The reconstructed networks can further help to compare the identified effects of environmental influence in healthy people with similar effects in patients with different forms of neuropsychiatric disorders of the depressive spectrum, and in non-clinical subjects with an increased tendency to develop depression or anxiety disorder, as well as to offer genetic mechanisms of these effects. Since gene networks are dynamic systems, their stable states (regimes of operation: stationary or cyclic) determine the state of electrophysiological activity of the brain and nervous system, and their response to the environment. In this regard, of particular interest are gene networks that work in different groups of people in the opposite (to some extent) way—depending on non-genetic factors. In this study, we have reconstructed a series of gene networks to build a model of the influence of complex interactions of environmental factors and genetic risk factors for intellectual disability, depression, and other disorders in human behavior. These are protein–protein interactions networks and networks of transcription regulation. We hence suggest a list of candidate genes whose expression is presumably associated with environmental factors and has potentially contentious manifestation for behavioral and neurological traits. Their further experimental verification might elucidate the possible mechanisms of development of anxiety, depression, and intellectual disabilities, and therefore make new treatment strategies.

## 2. Materials and Methods 

Genes that serve as the basis for the reconstruction of gene networks were selected by analyzing specialized data of genetic markers of human psychological diseases, such as: *SFARI* Gene [16], *BDgene* [3], *PsyGeNET* [17], *epiGAD* [18], *MK4MDD* [19], *HGNC* [20] and *PGC* [21].

The reconstruction of the gene networks was conducted using web services String DB [22]. String DB is a database of known and predicted protein–protein interactions. Interactions include direct and indirect associations; they are based on computer prediction and relationships collected from other (primary) databases. The network of manually selected genes was expanded using the Cytoscape StringExpand Network tool (http://apps.cytoscape.org/apps/stringApp), with Selectivity = 0.4. Analysis of enrichment of gene sets with terms of gene ontology (GO) was identified by DAVID 6.8 service [23]. All settings were used in the default mode.

### 2.1. Reconstruction of Transcriptional Regulation Networks

For all the genes from the sample, the coordinates of the exact location on the chromosome were obtained via Ensemble Biomart web service (GRCh38.p12 assembly of the genome was used). Promoter region sequences of length 300 b.p., 1000 b.p., and 2000 b.p. were extracted from the reference genome. Using AnimalTFDB database [24], we found transcription factors presented in our gene set (*TP53*, *SREBF2*, *SREBF1*). Using MoLoTool web-service, transcription factor binding sites (TFBS) were predicted for the three found transcription factors within upstream regulatory regions for all the genes. MoLoTool is an interactive web-based program to identify the sequence motifs of transcription factor binding for a given set of sequences [25]. It allows user to analyze up to 15 sequences at the same time, and gives the values of the TFBS significance. We used significance threshold *p_ajusted_* < 10^−4^, which is recommended by the web-service. Thus, the information about the hypothesized transcriptional regulation of genes inside a sample was received. Networks were visualized in Cytoscape [26].

### 2.2. SNP Analysis

We have analyzed SNP (single nucleotide polymorphism) distribution within both coding and promoter regions of genes. SNP analysis was performed to identify the most variable genes at the cross-section of the human population among the sample associated with intellectual disability depressive disorder. Such genes may have a more significant connection with the disorder due to the prevalence of their various variants in humans and, at the same time, with the high prevalence of the disorder itself in the population. SNP search was performed using 1000 Genomes [27] Project data with SAM Tools. Information on SNP distribution in coding and promoter (up to 2000 b.p. upstream) was extracted from the Project data, and then processed with Python scripts to summarize SNP enrichment for those regions. This information was finally superimposed on the networks. 

### 2.3. Comparison with Current Gene Panels

We have compared our gene networks with genes from commercial sequencing panels prepared by Roche, Illumina, and ThermoFisher for the study of neurological diseases (Appendix A). The gene sets covered by commercial panels from the Illumina (AmpliSeq for Illumina Neurological Research Panel, New-York, US) and ThermoFisher (Ion AmpliSeq™ Neurological Research Panel, Waltham, US) turned out to be identical, and therefore, we considered them as one set in the comparisons. Analysis of the overlapping of sets was performed using the tools of the Python programming language. The comparison is visualized using Venn diagrams.

## 3. Results

### 3.1. Formation of the Initial List of Genes 

To reconstruct the desired gene networks, we chose an initial set of genes. The main factor for choosing a gene was an ambiguity in phenotypic manifestation for the same variants in different human populations. Below there are six genes chosen as our initial gene set.

- *SLC6A4*—serotonin transporter gene. Many studies have shown a relationship between *5-HTTLPR* polymorphism and anxiety [2], depression, and bipolar disorder, antidepressant sensitivity [28], risk of depression, and suicide [29]. However, in a recent study, it has been shown to reverse the effects of this polymorphism in several Asian populations such as Japanese [12], Chinese [30], and Tuvinians [11].
- *HLA-B* (human leukocyte antigen) is one of the genes of the histocompatibility complex of class 1 in humans. Its allele, HLA-B *1502, plays an important role in the development of such carbamazepine-caused diseases as Stevens–Johnson syndrome and Lyell’s syndrome, but its effect is seen only in Chinese [31] and Malaysian populations [32]. In individuals of European origin, its influence was not found to be significant [33].
- *ABCB1* (ATP-binding cassette) is a gene of the membrane protein P-glycoprotein, which may transport xenobiotic substances through the cell membrane. *C3435T* allele of this gene supposedly plays a role in the development of drug resistance to epilepsy in a number of populations: Japan [34], Taiwan [35], but does not show a significant role in Korea [36], Turkey [37], Great Britain [38,39], and Ireland [40].
- Apolipoprotein E (*ApoE*) is one of the most important apolipoproteins, involved in the metabolism of blood lipids on the one hand, and cholesterol metabolism in the brain on the other. The *ε4* allele of this gene appears to be associated with an increased risk of temporal epilepsy after head injuries. Also, *ApoE* genotype is related to a level of intellectual disability, and presence of dementia in Down syndrome [41]. However, studies confirming this relationship were obtained only by researchers from the United States [42,43]. In other countries, no such relationship has been identified [44,45].
- *BDNF*—neurotrophin, a signaling protein that stimulates and supports the development of neurons. Peripheral *BDNF* level is associated with attention deficit and intellectual disability in preschool children [46]. In Japanese population, it is a trait of susceptibility to partial epilepsy [47], but in European populations, such dependence could not be detected [48,49].
- *COMT* is an enzyme that plays an important role in the degradation of catecholamines, including dopamine, adrenaline and norepinephrine and the role of *rs4680* polymorphism in the etiology of idiopathic intellectual disability was revealed [50]. Additionally, the role of *rs4680* as a risk factor for bipolar disorder was shown in Chinese population [51], as well as in Ashkenazi population [52], but its influence was considered insignificant in European populations [53].

### 3.2. Gene Networks Reconstruction

**Figure 1:**
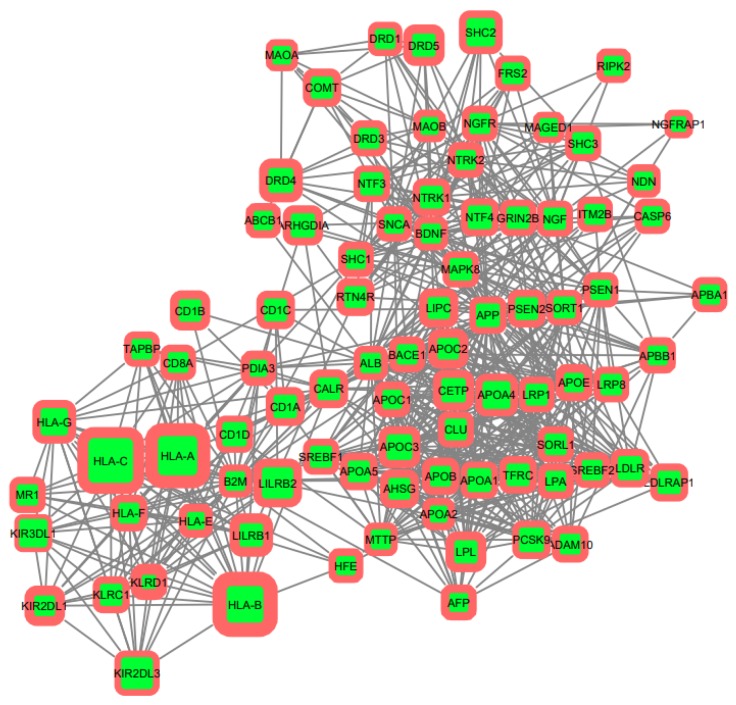
Protein–protein interactions network reconstructed on the initial gene set via String DB (visualized in Cytoscape). Size of node refers to SNP (single nucleotide polymorphism)-part in gene coding region, border thickness—SNP-part in promotor region.

Further, for the initial genes, the search of intergenic interactions was carried out via StringDB. According to the data obtained, reconstruction of protein–protein gene networks in the Cytoscape program was performed. Using the built-in String Expand tool from the String DB database, the resulting networks were built up with other proteins associated with our model (Figure 1).

### 3.3. Gene Sets Formation

From the reconstructed gene networks, a list of genes for sequencing was selected for further experimental validation of our model (Table S1). The list was analyzed using David GO web service. The results of the analysis indicate that our model is highly enriched with genes involved in neurophysiological and behavioral processes of neuropsychiatric diseases (Table 1).

### 3.4. Comparison with Current Gene Panels

**Figure 2:**
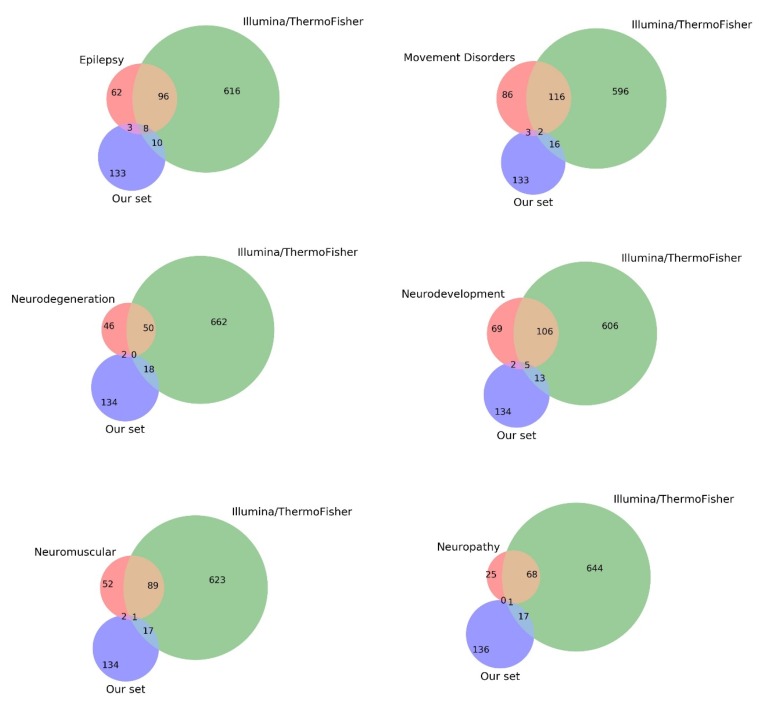
Venn diagrams of the overlapping of lists of genes, with lists of standard panels for target sequencing by Roche and Illumina/ThermoFisher.

Although the results of our screening do not overlap with the Roche panel (Figure 2), and Illumina/ThermoFisher panels (Figure 2), it is likely that this can be explained by the choice of genes: We prioritized the genes of neurological diseases with differences in phenotypic manifestation in different populations. Obviously, such a policy is not suitable for genes whose influence is modulated by environmental factors, which was our reason for not using standard panels for sequencing.

**Figure 3:**
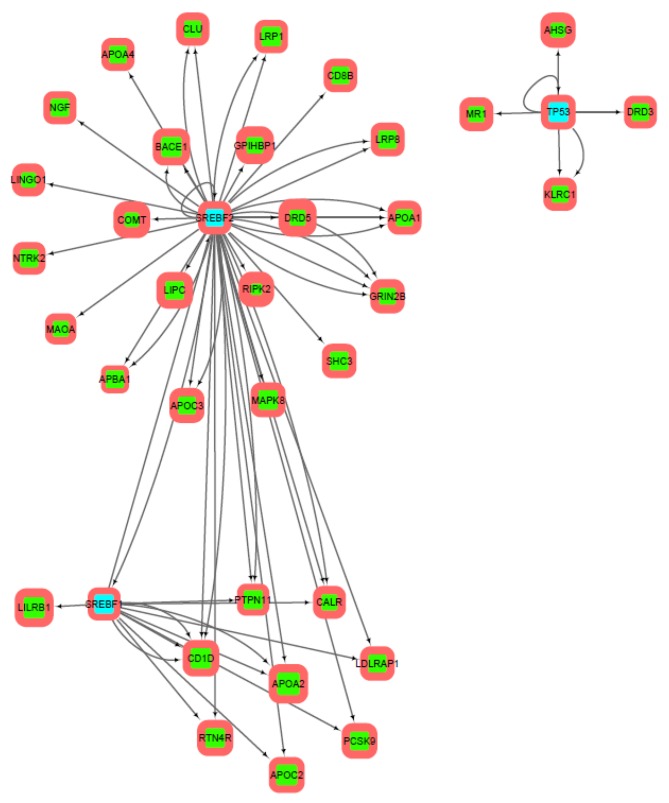
Transcriptional regulation network built on predictions of transcription factor binding sites in upstream promoter regions of 300 b.p. length. Transcription factors are cyan highlighted, green are regulated genes. Direction of arrows denote regulators (from) and regulated genes (to). The thickness of the frame corresponds to the enrichment of SNP in the upstream promoter region of the gene, the size of the node—the enrichment of SNP in the coding region of the gene.

The reconstructed network of transcriptional regulation built on upstream promoter regions of 300 b.p. length is shown in Figure 3. The least variable gene in it is *ARBA1* (0.7% SNP), the most variable is *APOA2* (4.5% SNP). Transcription factors *SREBF2* and *SREBF1* are combined into one cluster. It should be noted that the most part of genes (excluding *LILRB1*), controlled by *SREBF1*, have binding sites for *SREBF2*. At the same time, *SREBF2* regulates additional genes, which are not regulated by *SREBF1*. Moreover, *SREBF1* and *SREBF2* regulate each other. Transcription factor *TP53* form separate clusters with the four regulated genes: *DRD3*, *ahsg*, *mr1*, and *klrc1*.

**Figure 4:**
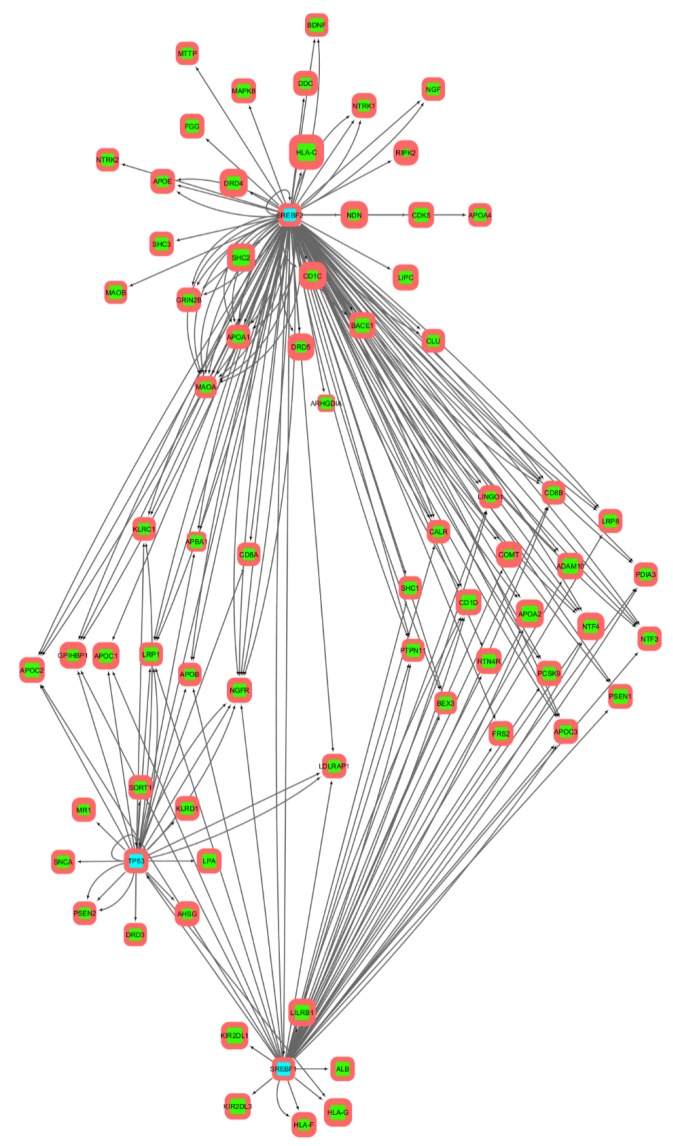
Transcriptional regulation network built on predictions of transcription factor binding sites in upstream promoter regions of 1000 b.p. length. Transcription factors are cyan highlighted, green are regulated genes. Direction of arrows denote regulators (from) and regulated genes (to). The thickness of the frame corresponds to the enrichment of SNP in the upstream promoter region of the gene, the size of the node—the enrichment of SNP in the coding region of the gene.

**Figure 5:**
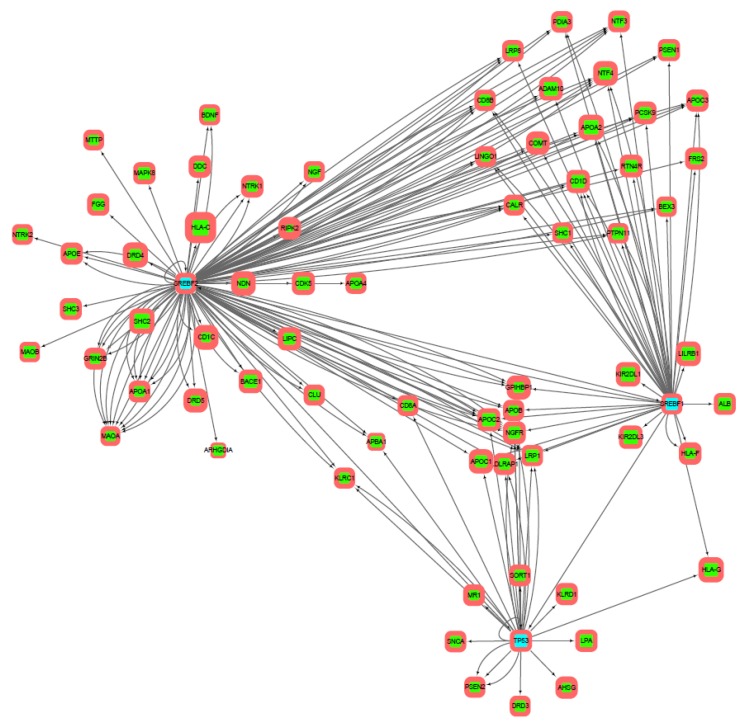
Transcriptional regulation network built on predictions of transcription factor binding sites in upstream promoter regions of 2000 b.p. length. Transcription factors are cyan highlighted, green are regulated genes. Direction of arrows denote regulators (from) and regulated genes (to). The thickness of the frame corresponds to the enrichment of SNP in the upstream promoter region of the gene, the size of the node—the enrichment of SNP in the coding region of the gene.

Networks built on upstream promoter regions of 1000 b.p./2000 b.p. (Figure 4 and Figure 5, correspondingly) look similar, so they will be described together. For each transcription factor, there is a set of genes regulated only by it, as well as clusters of genes regulated together with other factors. The largest number of regulated genes has *SREBF2*, the smallest—*TP53*. Among the genes constituting both the initial set of genes and the reconstructed gene networks, the highest share of SNPs is in those of the immune system (such as *KIR2DL3*, *LILRB2*, *HLA-A*, *HLA-B*, *HLA-C*). On the other hand, substitutions in the most conservative genes among the sample (for example, *BEX3*) can lead to more serious consequences for the functioning of molecular genetic systems. It is noteworthy that part of the genes (*BEX3*, *SHC2*) has not yet been studied, that is, there is no direct connection between not only molecular processes, but also phenotypes. Such genes are the main goal of our further experimental work. 

## 4. Discussion

We constructed the initial set of genes based on the principle of ambiguous phenotypic manifestation for the same polymorphisms for people from different populations, or for people exposed to different environmental influences. Resulted gene networks, constructed by adding genes functionally related to genes from the initial set, suggest hypothetical mechanisms for the realization of such a “contentious” phenotype. The fact that the vast majority of genes from gene networks are not contained in the diagnostic panels of psychiatric and neurological diseases, as well as behavioral disorders, suggests that these genes may be characterized by ambiguous phenotypic manifestation. This is supported by the GO enrichment analysis performed with the DAVID service—these genes are certainly associated with behavioral, neurological, and psychiatric disorders.

The next step of this research is to check the influence of the genes identified by us on such (endo)phenotypic characteristics as indicators of psychological tests, EEG parameters at rest, and under functional load on the brain and experimental behavioral data, determined for a sample of about 1000 people living in different regions of Russia and Mongolia. 

Analysis of transcriptional regulation networks (Figure 3, Figure 4 and Figure 5) built on information about TFBS distributions within upstream promoter regions of 300, 1000, and 2000 b.p. length has shown the 4 (in case of 300 b.p.) and 6 (in case of 1000 and 2000 b.p.) clusters of regulated genes. These clusters *(SREBF1-*, *SREBF2-*, *SREBF1/SREBF2-* and *TP53*-regulated genes in the first case and these plus pairwise regulated genes for the remaining pairs in the second case) may indicate several ways of transmission of transcriptional regulation signals, which also requires further experimental verification.

Thus, the genes found by us can become the basis for the formation of a new diagnostic panel to explain the “contentious” phenotypic manifestations and predict the impact of certain effects on their further manifestation.

## Figures and Tables

**Table 1:** GO enrichment analysis performed with DAVID GO for the formed gene set.

Category	Term	Count	Set Content (%)	*P* Value
GAD_DISEASE	Schizophrenia	59	3806	1.22 × 10^−33^
GAD_DISEASE	several psychiatric disorders	56	3612	1.51 × 10^−53^
GAD_DISEASE	Tobacco Use Disorder	53	3419	3.05 × 10^−4^
GAD_DISEASE	Type 2 Diabetes| edema | rosiglitazone	51	3290	1.293 × 10^−7^
GAD_DISEASE	Autism	46	2967	1.126 × 10^−35^
GAD_DISEASE	Bipolar Disorder	41	2645	2.60 × 10^−27^
GAD_DISEASE	alcohol consumption	35	2258	3.92 × 10^−38^
GAD_DISEASE	Alzheimer’s disease	27	1741	1.31 × 10^−5^
GAD_DISEASE	ADHD | attention-deficit hyperactivity disorder	24	1548	7.66 × 10^−24^
GOTERM_BP_DIRECT	GO:0007268~chemical synaptic transmission	38	2451	1.42 × 10^−35^
GOTERM_BP_DIRECT	GO:0007165~signal transduction	25	1612	9.60 × 10^−5^
GOTERM_CC_DIRECT	GO:0005886~plasma membrane	96	6193	7.52 × 10^−26^
GOTERM_CC_DIRECT	GO:0016021~integral component of membrane	81	5225	3.35 × 10^−10^
GOTERM_CC_DIRECT	GO:0005887~integral component of plasma membrane	63	4064	1.77 × 10^−29^
GOTERM_CC_DIRECT	GO:0070062~extracellular exosome	41	2645	4.12 × 10^−4^
GOTERM_CC_DIRECT	GO:0016020~membrane	37	2387	5.55 × 10^−5^
GOTERM_CC_DIRECT	GO:0005576~extracellular region	34	2193	1.15 × 10^−6^
GOTERM_CC_DIRECT	GO:0030054~cell junction	33	2129	1.23 × 10^−20^
GOTERM_CC_DIRECT	GO:0005615~extracellular space	28	1806	1.94 × 10^−5^
KEGG_PATHWAY	hsa04080:Neuroactive ligand-receptor interaction	51	3290	6.98 × 10^−40^
KEGG_PATHWAY	hsa04024:cAMP signaling pathway	26	1677	1.35 × 10^−15^

## Supplementary Materials

The following are available online at https://www.mdpi.com/2073-4425/10/9/699/s1, Table S1: List of genes selected for model validation.

## Appendix A

Neurology Research Panels Gene List Roche:

https://sequencing.roche.com/content/dam/rochesequence/worldwide/resources/brochure-seqcap-ez-neurology-panels-SEQ100290.pdf

Ion AmpliSeq™ Neurological Research Panel ThermoFisher:

https://www.ampliseq.com/tmpl/view.action?tmplDesignId=62969292#/?listAction=tmplCoverageSummaryList&tmplDesignId=62969292&wrapperId=ajaxTableWrapper

AmpliSeq for Illumina Neurological Research Panel:

https://www.illumina.com/products/by-brand/ampliseq/community-panels/neurological.html
